# Haploid genomes illustrate epigenetic constraints and gene dosage effects in mammals

**DOI:** 10.1186/1756-8935-6-41

**Published:** 2013-12-05

**Authors:** Martin Leeb, Anton Wutz

**Affiliations:** 1 Wellcome Trust-Medical Research Council Stem Cell Institute, University of Cambridge, Tennis Court Road, Cambridge CB2 1QR, UK; 2 Institute for Molecular Health Sciences, Swiss Federal Institute of Technology, ETH Zürich, Schafmattstrasse 22, Zürich 8049, Switzerland

**Keywords:** Haploid cells, Stem cells, Epigenetics, Development, Tumor, Evolution

## Abstract

Sequencing projects have revealed the information of many animal genomes and thereby enabled the exploration of genome evolution. Insights into how genomes have been repeatedly modified provide a basis for understanding evolutionary innovation and the ever increasing complexity of animal developmental programs. Animal genomes are diploid in most cases, suggesting that redundant information in two copies of the genome increases evolutionary fitness. Genomes are well adapted to a diploid state. Changes of ploidy can be accommodated early in development but they rarely permit successful development into adulthood. In mammals, epigenetic mechanisms including imprinting and X inactivation restrict haploid development. These restrictions are relaxed in an early phase of development suggesting that dosage regulation appears less critical. Here we review the recent literature on haploid genomes and dosage effects and try to embed recent findings in an evolutionary perspective.

## Review

### Haploid genomes in insects and mites

The information for the development of an organism is encoded in its genomic DNA sequence. In most animals each cell contains two copies of the genome making up a diploid chromosome set. Diploid genomes provide a buffer against deleterious effects of mutations and enable the maintenance of suboptimal alleles that could become advantageous if environmental conditions change. Furthermore, diploidy allows co-transmission of beneficial and suboptimal alleles from the same parent facilitating maintenance of a diverse genetic basis for selection to draw from. Advantages of diploidy might explain the rare observation of single copy genomes outside germ line development of animal species. In animal development, haploid genomes are largely limited to post-meiotic germ cells that show little proliferation and gene expression according to their specialized function in reproduction. Haploid genomes do occur in some social insects including ants, wasps and honeybees, where they determine male sex [1]. Haploidy presumably serves to purge deleterious mutations from the genome of males. Males are largely dispensable compared with females as they are only during a brief reproductive period in these species. A small number of fit males can provide a copy of the genome that is largely free of deleterious mutations for the next generation. In addition, rare parthenogenetic haploid species have been described in mites and insects [2,3]. Parthenogenetic all-female species appear to be rare exceptions but they still illustrate that haploid genomes can support development of quite remarkably complex organisms. Notably, haploid cell lines have also been isolated from flies that do not normally show haploid development [4] indicating that the ability to accommodate a change of ploidy is widely maintained in insect species. The scaling of molecular networks and pathways relative to genome copy number is surprising given the complexity of interactions involved in the animal developmental programs. Balancing of genome copy number elevations could be related to evolutionary selection for robustness of regulatory networks, but this has not been investigated to date.

### Limited haploid development in vertebrates

It is not hard to imagine that ploidy elevation can lead to problems for organismal development through different nuclear-cytoplasmic ratio or non-scaling gene dosage relations. However, evidence suggests that changes in genome copy number can be compatible with development in a range of organisms. Polyploid frogs and lizards can coexist with related diploid populations, and also interbreed in some cases [5-7]. A number of tetraploid amphibians and reptile species have been described [8]. Triploid vertebrates can arise through hybridization of diploid and tetraploid species or from nondisjunction of chromosomes in the egg after fertilization as is commercially applied in rainbow trout [9]. Even sexually reproductive triploid vertebrate species have been observed [7]. Notably, it has been possible to recreate ploidy elevation in the laboratory through fertilization of triploid eggs of parthenogenetic asexual vertebrates [6]. This suggests that little obstacles to ploidy elevation exist in vertebrates. Indeed there is evidence that two rounds of genome-wide duplications have occurred during vertebrate evolution [10,11], indicating that current vertebrate genomes are a relic from a polyploid stage [12]. Notably, haploid cell lines from frogs have been reported [13] showing that amphibians can accommodate both genome copy number elevation as well as reduction.

Haploid development in zebrafish can be experimentally induced by fertilization with inactivated sperm [14] or by fertilization of irradiated oocytes [15,16]. Haploid gynogenetic or androgenetic zebrafish embryos progress through embryonic development but do not reach the mature stage. This shows that in fish a haploid genome can direct embryonic growth and organogenesis but is incompatible with full adult development. Interestingly, haploid pluripotential embryonic cells from Medaka have been established [17]. These cells maintain an intact haploid karyotype in culture and can contribute to development through semicloning. Teleost fish have experienced a recent third genome duplication event and it is conceivable that haploid development could benefit from the approximation of an ancestral genome state before duplication [18,19]. These observations illustrate that developmental programs in fish, amphibian and reptile species can accommodate ploidy changes to variable degrees. It is conceivable that tolerance to ploidy changes is related to genome duplication events as a driver of evolutionary innovations in these branches [12]. Potentially more recent and complex developmental programs in higher vertebrates might introduce features that encounter greater problems with changes in ploidy.

### Imprinting and X chromosome dosage restrict haploid development in mammals

**Figure 1:**
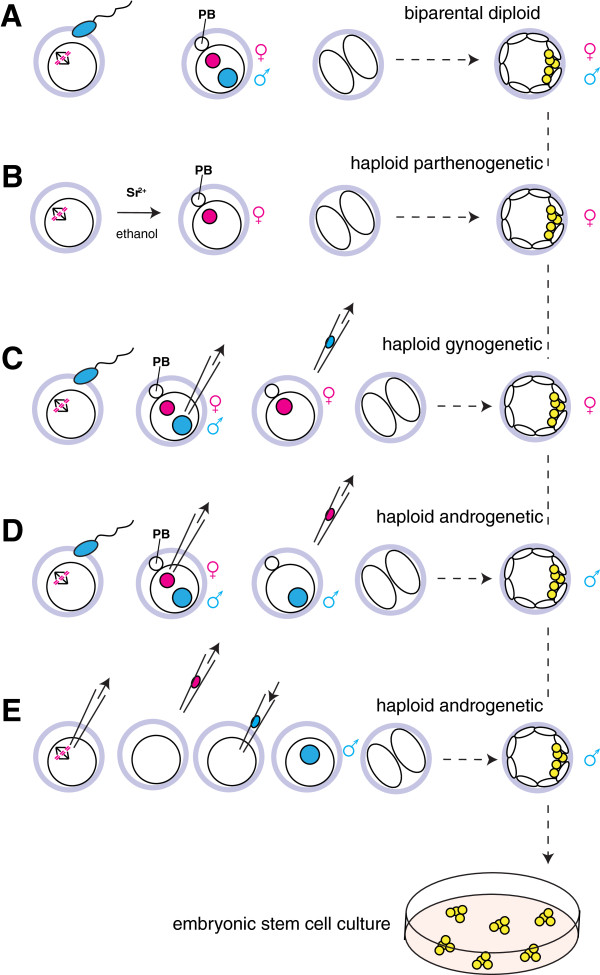
**Experimental production of haploid mammalian embryos. (A)** Normal fertilization results in embryos containing genomic contributions of both parents. During this process the metaphase II arrest of the oocyte is resolved and the second polar body (PB) is extruded leaving the diploid zygote with a haploid set of chromosomes from each parent. **(B)** Parthenogenetic activation of oocytes can be achieved by treatment with chemicals including Strontium salts or ethanol without fertilization and results in embryos that contain only one haploid set of maternal chromosomes [62,66]. **(C)** Similarly, haploid gynogenetic embryos can be constructed by removing the paternal pronucleus from a fertilized zygote by micromanipulation with a glass capillary in the presence of microtubule inhibiting chemicals. **(D)** Removal of the maternal pronucleus from the fertilized zygote results in androgenetic embryos containing only a haploid paternal genome [64,65]. Half of these androgenetic embryos containing the Y chromosome and lacking an X chromosome do not develop. **(E)** An alternative way for producing haploid androgenetic embryos is to enucleate the oocyte and introduce a sperm nucleus [64,65]. Between 10 to 20% of haploid embryos containing either the maternal or paternal set of chromosomes develop to the blastocyst stage when they can be used for establishing embryonic stem cell lines.

**Figure 2:**
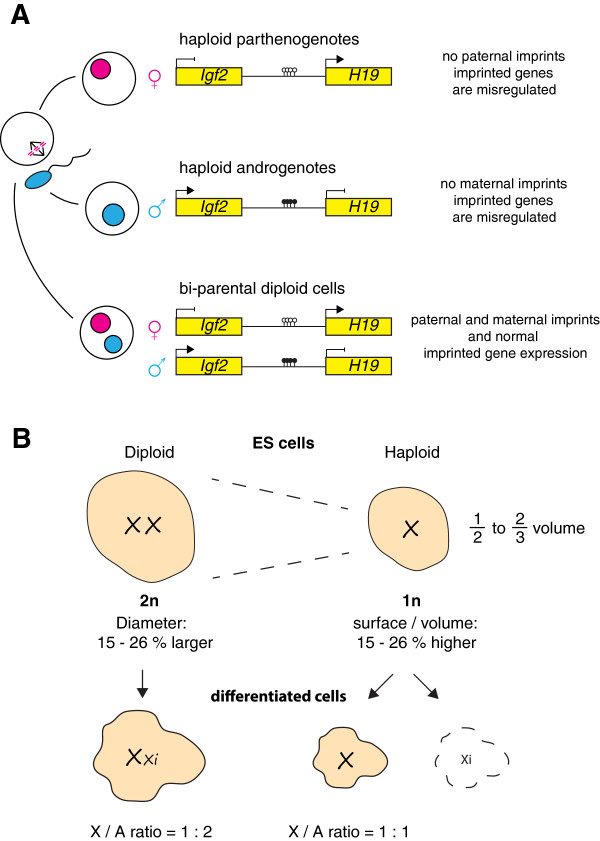
**Dosage imbalances in haploid mammalian cells. (A)** The inequality of parental genome contributions is illustrated by the *Igf2*-*H19* imprinted gene cluster. In bi-parental diploid cells, *H19* is expressed from the maternal whereas *Igf2* is expressed from the paternal inherited chromosome. Haploid cells only contain a single set of chromosomes, either the maternal or paternal, and therefore lack either *Igf2* or *H19* expression. **(B)** The cell volume of haploid cells is between 50 to 66% that of diploid cells. This leads to changes in the surface area to volume ratio and the cell diameter that can influence transport processes and extension of the mitotic spindle, respectively. In addition, dosage compensation by X inactivation is not feasible in a haploid karyotype and, as a consequence, a genetic imbalance is incurred as the X chromosome to autosome (X/A) ratio is elevated to 1:1 from 1:2 in normal diploid cells. This effect is only significant after embryonic stem (ES) cell differentiation as normal diploid ES cells are not dosage compensated by X inactivation.

In mammals, haploid development can be induced by activation of unfertilized oocytes to produce parthenogenetic haploid embryos [20-22] or by fertilization of enucleated oocytes to produce androgenetic haploid embryos [23] (Figure 1). Haploid mouse preimplantation embryos have also been obtained by mechanical bisection of zygotes [24] or by microsurgical removal of one pronucleus [25]. In mice, haploid cells have been observed until egg cylinder stage embryos [26], but haploid development beyond implantation is severely impaired. This is a direct consequence of the fact that the two parental contributions to the genome are not equivalent in mammals (Figure 2A). Genomic imprinting restricts expression of certain genes to one parental allele [27-29]. As a consequence, both maternal and paternal chromosomes are required for successful development in mice [30-32]. Genomic imprinting can affect evolution though selective exposure of mutations in a functionally hemizygous state and has further been suggested to aid a greater variability in quantitative traits that could benefit species in changing environments [33]. Fixation of unbalanced parental contributions is hypothesized to be driven by genetic effects including conflict over parental investment between both sexes [34,35].

Other examples for monoallelic expression in mammals include allelic exclusion of immunoglobulin loci [36], T-cell receptor genes and olfactory receptor genes. In addition, the majority of X-linked genes are expressed monoallelically. Compensation for X-linked gene dosage is required as a consequence of the mammalian XY sex chromosome system. In both males (XY) and females (XX), only a single X chromosome is transcriptionally active [37]. This is achieved by transcriptional inactivation of one of the two X chromosomes in females through the process of X inactivation. The requirement of a single active X chromosome per diploid set of autosomes results in an X chromosome to autosome ratio of 1:2 that cannot be approximated within a haploid genome and causes immitigable dosage effects for haploid development in mammals (Figure 2B). Gene activity from the single X chromosome causes a two-fold relative increase in X-linked gene dosage. Alternatively, inactivation of the X chromosome leaves haploid cells nullisomic for X-linked genes, which is not compatible with survival [38]. Whereas early mouse embryos can tolerate a lack of dosage compensation, X inactivation becomes essential soon after implantation [39]. Genomic imprinting, monoallelic expression and X chromosome dosage impose genetic limits to haploid development in mammals.

### Haploid phases in human tumors

It is a fact - despite rarely being consciously considered - that a diploid karyotype represents an exception rather than the rule in established cell cultures. Many permanent cell lines acquire aneuploidies in culture with gain and loss of chromosomes providing growth advantages possibly in combination with acquired mutations. Culture conditions might contribute significantly to the development of aneuploidies as growth requirements are less stringent than in development where growth depends on functioning tissues and organs. This is also true for mouse embryonic stem (ES) cells where aneuploidies accumulate with an increase in passage number [40]. Notably, aneuploidies are also observed in rare occasions of transmissible tumors in canines and Tasmanian devils suggesting that unusual and unexpected properties can result from karyotype changes [41,42]. Elevated levels of aneuploidy are also common in human tumors. These observations suggest that a diploid chromosome set is not essential for cell survival and deviations from a regular diploid genome might be advantageous in culture and tumors.

**Figure 3:**
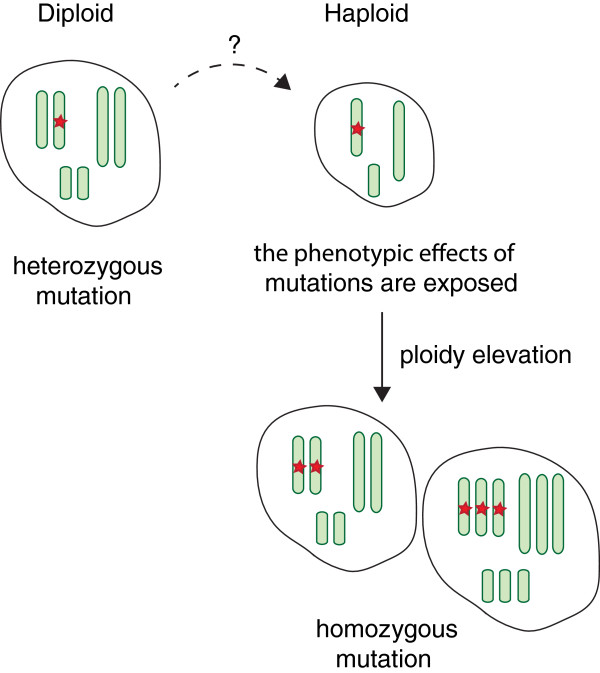
**Haploid phases are observed in human tumors.** Haploid phases in human tumors could facilitate or accelerate the loss of tumor suppressor gene function. Mutations that have been introduced into the haploid tumor genome will become homozygous when the tumor cell becomes diploid or polyploid. The observation of tumors with cells at various polyploidy levels can follow a transient haploid phase, which makes recognition of haploid phases difficult.

Aneuploidy in most tumors manifests itself in a shift of the modal average of chromosomes. Interestingly, hypodiploid, including rare near haploid tumor karyotypes, have been reported. Near haploid tumor cells have been observed in rare cases of leukemia [43-49], and have been less frequently reported in solid tumors [50-52]. Loss of chromosomes appears to be the primary event in near haploid acute lymphoid leukemia and correlates with poor prognosis [44,53]. Haploid karyotypes in tumors are not fully intact and often contain diploid genomic regions and chromosomal rearrangements [54]. This suggests a selective advantage of the haploid state, probably in the context of oncogenic mutations and rearrangements. A haploid phase where a single hit can inactivate gene function could be explained by selection for loss of tumor suppressor genes during tumor development (Figure 3). However, it is unlikely that haploidy is a requirement for loss of tumor suppressor activity since this could also be achieved by selective loss of few chromosomes and maintenance of a largely diploid genome. Therefore, it cannot be ruled out that a haploid phase might contribute to tumor cell persistence in a different way, possibly involving gene dosage effects.

### Establishment of haploid mammalian cell lines

Cells with near haploid and hypodiploid karyotypes have been adapted to growth in culture from a partially haploid chronic myeloid leukemia [43]. Apparently, these cultures were obtained at the blast phase after a long benign phase and repeated chemotherapeutic treatment suggesting significant selection of tumor cells before cultures were established. The haploid portion of the KBM7 cell line carries two copies of chromosomes 8 and 15 in addition to a BCR-ABL chromosomal translocation. Initially, the KBM7 cell line showed strong inclination to diploidization such that later passages had lost the haploid fraction of cells. However, a subclone (P1-55) from early passage KBM7 cells maintained a near haploid karyotype diploid only for chromosome 8 stably in culture [47]. Notably, the reduced rate of diploidization indicates a second and independent adaptation that has occurred after culture. Later work has attempted to change the cell type of the haploid cells for expansion of their use in genetic screening [55]. Introduction of viral vectors used for reprogramming of induced pluripotent stem cells resulted in an adherent cell line that had lost its hematopoietic character. Although pluripotency was not established, these HAP1 cells are of interest as they possess different growth properties including altered morphology and differential response to cell toxins [55]. This cell line also no longer contains a second copy of chromosome 8 suggesting a haploid karyotype, albeit with chromosomal translocations. These changes have also led to an increased rate of diploidization. These findings clearly illustrate that mammalian cells with a near haploid karyotype can proliferate and display distinct phenotypes in culture.

### Pluripotent haploid cells from early mouse embryos

Following studies on haploid mammalian embryos, initial attempts to derive pluripotent ES cells from haploid mouse blastocysts resulted in the establishment of diploid cell lines [56]. This was surprising as both parthenogenetic and androgenetic diploid embryos can develop past the blastocyst state and survive beyond implantation [57,58]. Parthenogenetic embryos are lost around embryonic day 10 (E10) [58,59]. Similarly, embryos with impaired dosage compensation due to a mutation in the *Xist* gene develop beyond implantation [39,60]. These findings indicate that pre-implantation development is largely independent of dosage compensation and the presence of a bi-parental complement of imprints. However, pre-implantation development in parthenogenotes does not progress completely independent of X inactivation and delayed upregulation of *Xist* from one of the two maternal X chromosomes has been reported at the eight cell stage [61]. Recent improvements in ES cell culture techniques and innovation in flow cytometric cell sorting technology have finally facilitated the establishment of haploid parthenogenetic [62,63] and androgenetic [64,65] ES cell lines from mouse embryos (Figure 1). Haploid mouse ES cells proliferate in culture and maintain an intact haploid karyotype for more than 30 passages as evidenced by genomic analysis and developmental competence [66].

The developmental stage from which mouse ES cells are derived appears to tolerate the loss of epigenetic regulation [67]. It has been reported that abrogation of DNA methylation [68], Polycomb complex function [69] and nuclear B type lamins [70] does not prevent proliferation and self-renewal of mouse ES cells. In contrast, respective mutations lead to defects in differentiated cells. ES cells are derived from cells of the inner cell mass of the blastocyst that will develop into the epiblast. At these stages epigenetic patterns are reset and epigenetic regulation appears substantially different. For example, the cells of the early epiblast are not dosage compensated before X inactivation is initiated around the time of gastrulation in mice. The discovery of new culture conditions has facilitated the culture of ES cells in a naïve pluripotent ground state by inhibition of the mitogen activated protein (MAP) kinase and glycogen synthase kinase pathways [71]. These two inhibitor (2i) conditions are beneficial for obtaining ES cell lines with a high content of haploid cells [63]. Haploid ES cells have also been established or cultured in traditional serum containing media and Leukemia inhibitory factor (LIF), but with substantially reduced efficiency and increased rate of diploidization [62,66]. The question arises how 2i culture conditions contribute to the maintenance of a haploid karyotype. In serum-based culture conditions, ES cells are heterogeneous and at any given point in time only a fraction of cells express naïve pluripotency markers including Nanog and Rex1. In contrast, these markers are homogenously expressed in all cells in 2i conditions [71-73]. Therefore, it is conceivable that, in the naive ground state, selective pressure arising from gene dosage effects of a haploid genome are largely alleviated. Notably, culture in 2i medium also induces drastic changes in the epigenetic profiles of ES cells. It has been shown that Polycomb-associated histone H3 tri-methylation patterns are shifted in 2i conditions with reduced levels on promoters and greater enrichment over satellite repeats [74]. Furthermore, DNA methylation is substantially reduced in 2i medium compared to serum-based ES cell cultures [75]. This finding is consistent with low levels of DNA methylation in inner cell mass cells. Irrespective of reduced epigenetic modifications, genomic imprints are maintained in 2i medium [75]. Paternal imprints are further partially maintained in androgenetic haploid ES cells but are progressively lost with time in culture [64,65]. Loss of imprinting is not special to haploid ES cells but can also be associated with diploid ES cell cultures [76]. Haploid ES cells are competent to contribute to a wide range of tissues in chimeras [62-65]. However, contribution to development is only possible after diploidization *in vitro* or *in vivo*. Colonization of the female germ line and transmission of a transgene was observed for parthenogenetic haploid ES cells [66]. So far, the germ line competence of androgenetic haploid embryonic stem cells has been limited to early stages of primordial germ cells [64,65]. However, they have been used for semi-cloning and appear to contain functionally relevant paternal imprints. Live mice have been obtained from injection of haploid androgenetic ES cells into unfertilized oocytes indicating that they can substitute sperm cells [64,65]. Haploid ES cells appear to have an intrinsic tendency for diploidization. The trigger for diploidization is not known but appears crucial for the establishment of stable differentiated cell types from haploid ES cells. Haploid ES cells proliferate with similar kinetics as diploid ES cells. Hence the need to replicate a genome that is half the normal size does not appear to increase proliferative potential. This observation is further in line with the observation that tetraploid ES cells show a similar rate of self-renewal as diploid ES cells [77]. This indicates that the replication of the genome is not the rate limiting step in cell division in ES cells. Alternatively, altered properties might compensate for differences in genome size. Interestingly, ploidy correlates with cell size. Haploid ES cells possess a volume that is approximately two-thirds of that of diploid cells (unpublished observation; Figure 2B). Therefore, a reduction in genome size leads to a concomitant reduction in the availability of resources due to a smaller cell volume.

### Application of haploid cells in genetic screens

**Figure 4:**
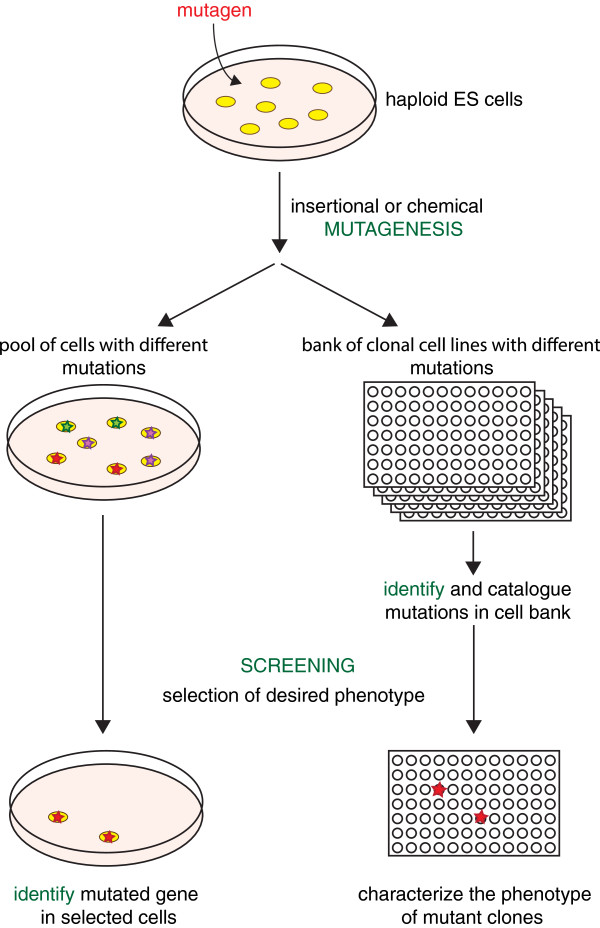
**The use of haploid cells in genetic screening.** A primary interest in haploid cells is their use for generating mutations for assignment of gene function. In haploid cells, loss of function mutations can be readily generated as no complementation by the homologous chromosome set is encountered. Phenotypic exposure to various selection strategies can be used to investigate gene function in specific pathways. Alternatively, libraries of cells containing mutations in genes can be generated and characterized. Screening in cell culture is a distinct advantage in mammals where combination of mutations to homozygosity requires breeding efforts that are both costly and time consuming. ES, embryonic stem.

Diploid karyotypes of virtually all mammalian species have severely limited forward genetic approaches. The discovery of haploid mammalian cells has opened new possibilities for performing genetic screens in mammals (Figure 4). The use of haploid cells in screens was initially demonstrated using a near haploid KBM7-derived human tumor cell line [78]. For this a mutant library was generated by insertion of viral gene trap vectors in large pools of haploid cells. This library was subsequently exposed to various toxins and pathogens. Surviving cell clones were analyzed and mutations conferring resistance could be identified. Since then an impressive number of screens investigated host mechanisms utilized by pathogens [55,78-82]. In addition to understanding disease mechanisms, recent work has also provided insights into human cellular pathways [81]. Haploid ES cells could provide advantages through a largely intact genome that is free of tumor-specific mutations. This is especially important for dissection of developmental processes in forward genetic screens. The potential for using haploid ES cells in screens has been shown by proof-of-principle experiments identifying factors in the DNA mismatch repair pathway and mediating ricin toxicity [62,63]. Inherent tendency to diploidization is not an obstacle for screening as long as the mutations are inserted in a haploid state. Recently, haploid ES cells have also been used to investigate the resistance mechanism for the chemotherapeutic agent Olaparib [83]. It might be expected that future screens can utilize the pluripotent potential of haploid ES cells and the availability of reporter mouse lines for investigating molecular networks of gene regulation, cell signaling and development. This prospect suggests that haploid ES cells could become a tool for performing developmental screens in culture similar to screens in haploid zebrafish [84].

## Conclusions

The ability to derive haploid ES cells might be facilitated by a distinct developmental state. Epigenetic mechanisms are largely dispensable in preimplantation mouse embryos. This likely reflects a period of resetting the genome to attain pluripotency. In addition, cell size in preimplantation embryos changes in a remarkable fashion through successive cleavage divisions of the oocyte during which overall embryo growth is negligible. This leads to progressively smaller cell sizes. It is conceivable that regulatory networks have been adapted to cope with changing cell sizes and, thus, are robust against dosage effects. Indeed, ES cells appear to tolerate considerable changes in gene expression profiles. Gene expression profiles in Polycomb-deficient ES cells are substantially changed but do not abrogate self-renewal [69]. Notably, a recent comparison of serum and 2i culture has also identified surprisingly large differences in gene expression [74]. These observations suggest that regulatory networks in ES cells are robust to disturbances in gene expression patterns. This robustness could contribute to the scaling of pathways with different levels of ploidy.

The extent to which differentiated cell types can be maintained with a haploid karyotype remains unknown. Induction of haploid ES cells to differentiation conditions inexorably leads to rapid diploidization. An indication that haploid karyotypes are compatible at least with early developmental cell fates comes from reports showing that haploid epiblast stem cells and primitive endoderm-like cells have been established from haploid ES cells in culture [64,66]. These reports are consistent with the observation that haploid cells can contribute to E6.5 post implantation embryos before diploidization [63-65] and have been observed in egg cylinder stage embryos [26].

Development of haploid embryos is affected by requirements for imprinted gene expression and dosage compensation. Haploid ES cells can contribute to the development of chimeric embryos after diploidization but are unable to support ES cell derived mice in a tetraploid complementation assay [66]. Imprinting defects are illustrated by the inability of diploid parthenogenotes to progress through development beyond E10 [59]. Interestingly, it has been possible to generate bimaternal embryos that can develop normally from fully grown oocytes and non-growing oocytes that contain double deletions in the H19 differentially methylated region and the Dlk1-Dio3 intergenic germ line-derived imprinting control region [85]. It is interesting to consider if similar manipulations could improve the stability and differentiation potential of parthenogenetic haploid cells. The imprints that inhibit androgenote growth are yet to be determined.

The dosage compensation problem is more difficult to resolve as a half dose of X chromosome linked genes would be required in the case of a single set of autosomes (Figure 2A). The relative expression balance for X-linked and autosomal genes is assumed to be maintained in evolution through upregulation of the active X chromosome relative to autosomes following Y chromosome erosion and a switch to a single active X chromosome [86]. The mechanism of X upregulation is presently not well understood. Recent results suggest that the Males absent on the first (MOF) histone acetyltransferase contributes to the upregulation of a subset of X-linked genes [87]. Interference with the mechanism of X upregulation could potentially be considered for reducing the X-linked gene dosage in haploid cells. Not all X-linked genes appear to be upregulated and subject to dosage compensation [88]. Expression reduction, possibly by RNAi-mediated strategies, could therefore also be considered. Genes whose products contribute to multi-subunit complexes appear most critical, as loss of stoichiometry can topple the balance of fine-tuned regulatory networks and protein-complex formation [89-91]. Restoration of X dosage, and hence stoichiometry, could be an effective means for enhancing haploid cell stability and developmental performance.

The observation of haploid phases in human tumors suggests that certain oncogenic signals can stabilize a haploid karyotype. Notably, overexpression of X linked genes has been implicated as a driver of tumorigenesis [92,93]. Future work will be needed to establish a connection between oncogenic transformation and changes in ploidy. This could yield important insights into dosage sensitive pathways in mammals and also be relevant for understanding certain human tumors. Dosage balance is less critical in differentiated cells and aneuploidies are tolerated in tumors and cell cultures to some extent. Dosage regulation could be critical in a developmental window but be less stringent in preimplantation development and at the end of the developmental program. An interesting question is if haploid cells can be generated directly from somatic diploid cells. Loss of chromosomes has been experimentally induced by interfering with centromere function [94]. Loss of chromosomes often appears to lead to aneuploidies that are not compatible with cell survival and proliferation. It appears that, in contrast to tumor cells, relative gene dosage imbalances are more detrimental to survival of untransformed cells than haploidy. This suggests that reduction of a diploid to a near haploid karyotype in a single instance or rapid succession of manipulations would be required. It is hard to imagine how this could be achieved with current technology. Induction of meiosis could in principle be considered as an alternative strategy. However, meiosis is an elaborate process that requires pairing of homologous chromosomes which in animals has not been observed outside the germ line. Recent advances in culture systems suggest that the generation of germ cells might become feasible. Protocols for deriving oocytes [95,96] and sperm [97] from ES cells have been reported. These methods could be useful for establishing haploid cells from ES cells or germ line precursor cells. Lastly, the still elusive mechanism that cancer cells use to reduce the genome by half might be applied for experimental induction of haploidy in cell cultures. Undoubtedly, future research will contribute to methods for establishing haploid cells and rebalancing gene dosage that could finally lead to an increased developmental potential.

Independently, haploid ES cells might provide a tool for studying allelic differences in genomic imprinting. The ability to establish haploid androgenotes and parthenogenotes will allow the maintenance of the two parental genome contributions in separate cell cultures and facilitate the functional investigation of parental marks. Although the haploid cell state is, with the exception of gametes, either artificial or associated with malignancies, it holds the promise of teaching us about genomic balance and dosage effects. Haploid embryonic cells will have important implications for understanding gene regulatory networks and genome evolution and will provide a powerful genetic screening platform.

## Abbreviations

2i: Two inhibitor; E10: Embryonic day 10; ES: Embryonic stem.

## Competing interests

The authors declare that they have no conflict of interest in writing this review, but wish to make it known that a patent application covering haploid embryonic stem cells has been filed (patent publication number WO2012117254 A1).

## Authors’ contributions

ML has researched the topic and written the text of the review. AW has written the text and advised on the content and structure of the review. Both authors read and approved the final manuscript.
